# Management Modalities of Primary Bladder Neck Obstruction in Young Adult Men: A Systematic Review and Meta-analysis

**DOI:** 10.5152/tud.2024.23155

**Published:** 2024-01-01

**Authors:** Ankur Mittal, Gurpremjit Singh, Vikas Kumar Panwar, Sanjay Sinha, Arup Kumar Mandal

**Affiliations:** 1 All India Institute of Medical Sciences, Uttarakhand, India; 2 Apollo Institute of Medical Sciences and Research Hyderabad, Telangana, India

**Keywords:** Bladder neck obstruction, lower urinary tract symptoms, primary bladder neck obstruction, young men with lower urinary tract symptoms

## Abstract

Objective: This systematic review was done to critically appraise the various evidence available in the literature for the presenting symptoms, diagnosis, and management modalities for primary bladder neck obstruction diagnosed on invasive urodynamics in young adult men 18-50 years of age.

Methods: A search was conducted on PubMed, Embase, and Cochrane Central Register of Controlled Trials databases until July 2022 to find English-language studies relevant to the topic.

Results: A total of 10 studies were included. The estimated difference in International Prostate Symptom Score between baseline and 3 months in the subgroup of medical and surgical treatment was found to be −8.82 and −11.25, respectively (*P *= .37), and after 12 months, it was found to be −7.69 and −17.70 respectively (*P *< .001). The pooled estimate for the difference in Qmax between baseline and 3 months after medical and surgical treatments in the subgroup was found to be 2.92 and 7.03, respectively (*P *= .18), and after 12 months, it was found to be 4.54 and 7.74, respectively (*P *< .001). The pooled estimate of the difference in post-void residue before and after 3 months of medical and surgical treatments in a subgroup was found to be −31.15 and −70, respectively (*P *< .001), and after 12 months, it was found to be −31.49 and −156.00, respectively (*P *< .001). Quality of life scores improved in both subgroups.

Conclusion: The alpha-blockers are effective in managing primary bladder neck obstruction in the short term, while bladder neck incision is preferred for better long-term outcomes.

**Main Points**

- Before providing PBNO patients with an acceptable treatment option, it is essential to consider their preferences and subjective response rate.
- Alpha-blockers can be considered for short-term treatment.
  Bladder neck incision has better long-term results

## Introduction

Bladder outlet obstruction in men can be caused by primary bladder neck dysfunction (PBND)/primary bladder neck obstruction (PBNO) in younger patients or benign prostate enlargement in older patients.^1^ Marion first used the term in 1933.^2^ Turner-Warwick and colleagues described primary bladder neck dysfunction as a non-neurogenic condition in young men. The theory behind bladder neck dysfunction includes an abnormal arrangement of musculature in the bladder neck region. Detrusor muscle contraction causes narrowing of the bladder neck instead of funneling and leads to functional obstruction.^3,4^ The importance of urodynamics (UDS) in diagnosing bladder neck dysfunction in young males is well advocated.^3^ Norlen and Blaivas used video urodynamics (vUDS) criteria to diagnose primary bladder neck obstruction, including high pressure voiding, low flow rate on uroflowmetry, and bladder neck narrowing with sphincter silence on electromyography.^5^

Initial therapy for men with voiding lower urinary tract symptoms (LUTS) is typically based on a clinical diagnosis and consists of alpha-adrenergic blockers. The follow-up for these patients is inadequate and often leads to frustrating outcomes. Most of these patients might not undergo a precise urodynamics diagnosis. Hence, their clinical outcomes remain unclear. Invasive urodynamics is typically recommended for young men under 50 years of age for whom invasive therapy is being considered.^6^ These are men who are refractory to conservative treatment or have red flags at the outset, such as hydronephrosis, necessitating urgent intervention.

This systematic review and meta-analysis aims to evaluate the available evidence on presenting symptoms, diagnosis, and treatment modalities for PBNO. Our research is novel because it exclusively examines primary bladder neck obstruction in young adult men, a group that has frequently been ignored in previous studies, and because it thoroughly synthesizes the data, offering a complete insight into this disease.

## Material and Methods

A systematic review was conducted using inclusion and exclusion criteria to gather relevant data. The institutional research review board duly approved the protocol. The International Prospective Register of Systematic Reviews (PROSPERO) registration number was CRD42021216042. The Preferred Reporting Items for Systematic Review and Meta-Analysis (PRISMA) checklist was used for reporting this review.^7^ The Institutional Ethics Committee approved the review.

### Date Resources and Searches

The review followed the PRISMA and MOOSE guidelines for conduct and reporting.^7^ A systematic search was conducted in PubMed, Embase, and Cochrane to identify relevant English language studies published until July 2022. An additional hand search was conducted by examining other sources, such as citations in appropriate articles. Supplementary Table 1 gives the search strategy. The terms used for the search were primary bladder neck obstruction, primary bladder neck dysfunction, and bladder neck obstruction.

### Eligibility Criteria

We enrolled male subjects aged 18-50 years with primary bladder neck obstruction identified through urodynamic evaluation. Studies reporting data for heterogeneous groups that do not specify an outcome for young men with PBNO separately were excluded from the analysis. Given our preference for peer-reviewed articles and quality control, we chose to avoid using gray literature.

### Study Selection and Data Extraction

After removing duplicate studies, the first reviewer evaluated the titles and abstracts, which the second reviewer cross-checked. The relevance of each work was assessed following the inclusion criteria. The second reviewer and the supervisor piloted the study screening tool. Any disagreements were handled either by consensus or after discussion with the supervisor. The full text of the selected studies was evaluated to determine whether they were eligible to be included in the review. The PRISMA flow diagram in the results section depicts the steps followed for selecting eligible studies. An independent data extraction proforma, created in advance by the first reviewer, was used to collect data and then cross-checked by the second reviewer. Following the characteristics described in the included research, modifications and adaptations to the proforma were made at various stages of the extraction process. In addition to study design and sample size, information was also collected on publication year and outcome measures.

### Evidence Quality and Bias Risk Assessments

The Methodological Index for Non-Randomized Studies (MINORS) was utilized to assess bias risk in this review. The MINORS scale has^8^ methodological criteria for non-comparative, non-randomized research and 4 extra criteria designed explicitly for comparative, non-randomized studies. The data is assigned a score of 0 when it is not reported, a score of 1 when it is reported but deemed poor, and a score of 2 when it is reported and considered adequate. The index demonstrates strong inter-reviewer agreement, satisfactory internal consistency, and robust test–retest reliability. Research studies that obtained MINORS scores of more than 50% were regarded as excellent quality. Nevertheless, due to the nature of the studies included in this review being descriptive cohorts, it was necessary to make suitable modifications to the MINORS scale.

### Data Analysis Including Statistical Analysis

For all studies covered, narrative data synthesis was carried out. The study characteristics table under the results section presents critical facts about each study.

To ensure consistency, we reviewed the studies for homogeneity. Statistical heterogeneity was assessed using the *I* ^2^ statistic and the Cochrane Q test, and the Comprehensive Meta-Analysis software was used to create forest plots for various outcome measures. We used a random-effects model, and the forest plots were accompanied by the *I* ^2^ statistic values and 95% confidence intervals, depicting high heterogeneity among the studies. Appropriate statistics were used to report different outcome measures, such as mean and standard deviation, median and interquartile range for continuous variables, and proportions or percentages for categorical variables. Other relevant statistical parameters based on the available data were also reported. We calculated the standard deviation for studies that presented means with standard error or confidence intervals to determine the pooled estimates. The alpha significance level for the statistical test was set at 0.05.

## Results

### Literature Search

**Figure 1.**
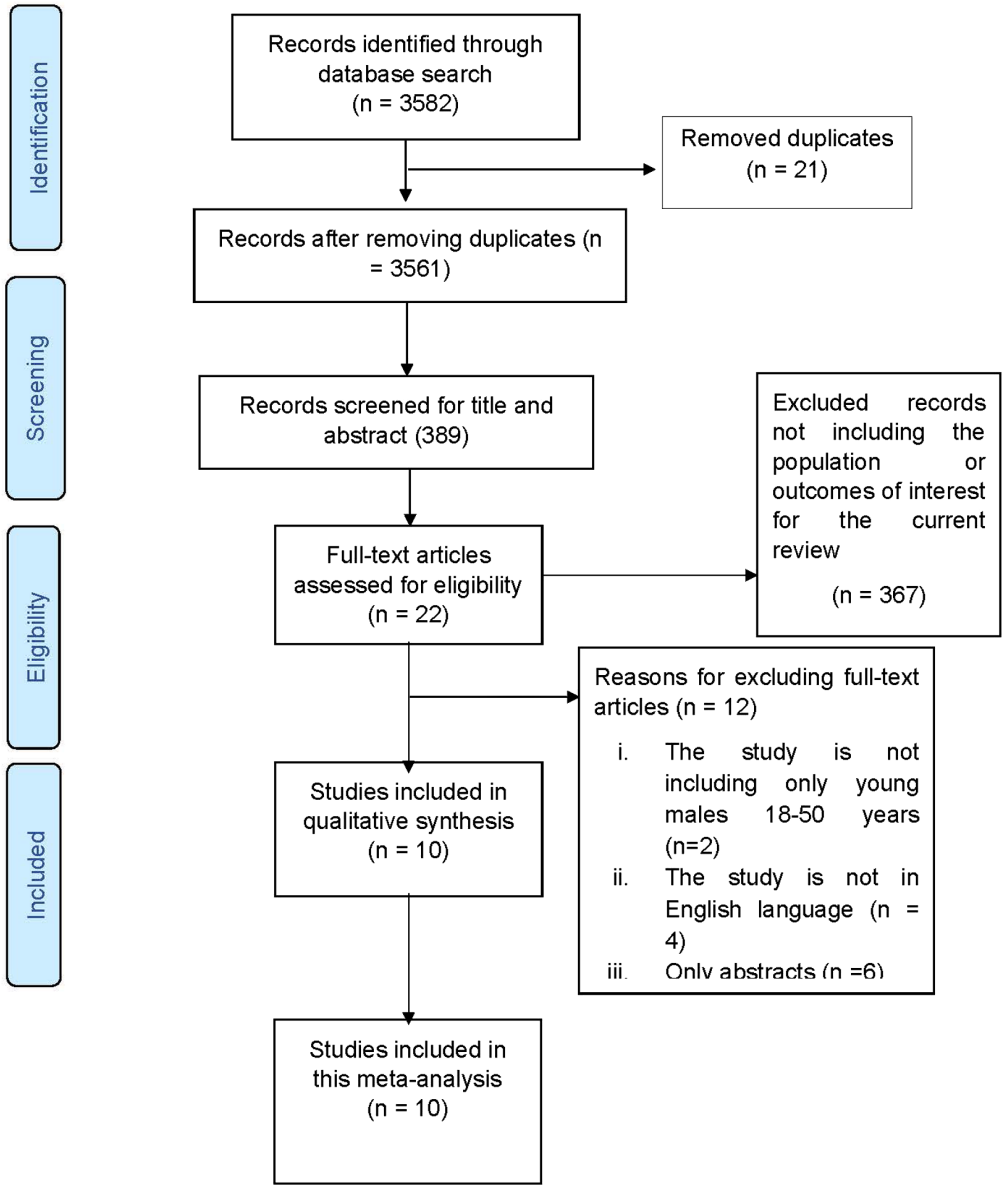
Preferred Reporting Items for Systematic Review and Meta-Analysis flow diagram for selection of eligible studies.

After searching all accessible databases, 3582 studies were found. After removing duplicates, 389 studies were screened. Title and abstract screening were conducted for 22 studies, and 10 met the inclusion criteria. A manual search was conducted to find any missing research in the references of each included study. Ten studies were ultimately included in this systematic review and meta-analysis. The study by Suri 2005^9^ et al had subgroups of medical and surgical management, so they have been divided accordingly for meta-analysis. The PRISMA flowchart is given in Figure 1.

### Overall Study Characteristics and Characteristics of Individual Studies

This systematic review analyzed data from 10 studies involving 495 men aged 18-51, published between 2000 and 2020, and with various age groups of subjects (Table 1). All patients were diagnosed with PBNO. The average symptom duration was 35.9 months. The clinical presentation of various patients, as described in the numerous studies, is mentioned in Table 1. Primary bladder neck obstruction diagnosis was established using vUDS in all the studies. The various treatment options and success definitions used in the different studies are given in Table 2. The quality-of-life scoring data was heterogeneously described in numerous studies. It has been summarized in Table 3. The adverse events associated with the treatment options used are given in Table 2.

### Risk of Bias

The risk of bias as determined by the methodological index for non-randomized studies (MINORS) (9) is shown in Supplementary Table 2. The MINORS scale was modified according to the studies in this review. An intermediate risk of bias was present in 3 studies, and a low risk of bias was present in 7 studies. The Oxford level of evidence in the studies was also assessed. Level 2B studies were 7, and Level 4 studies were 3.

### The Success Rate of Various Treatment Modalities

Three variables were used for the success rate of various treatment modalities: International prostate symptoms score (IPSS/American Urological Association symptoms score, maximum flow rate (Q_max_) max, and post-void residue (PVR).

The meta-analysis is conducted based on these 3 variables according to the follow-up time available in the studies for comparison. The meta-analysis is divided based on medical management, surgical management using bladder neck incision, or onabotulinum toxin A (OnaBoNT-A).

#### 1. The Change in International Prostate Symptoms Score Between Baseline, 3 Months, and 12 Months:

**Figure 2:**
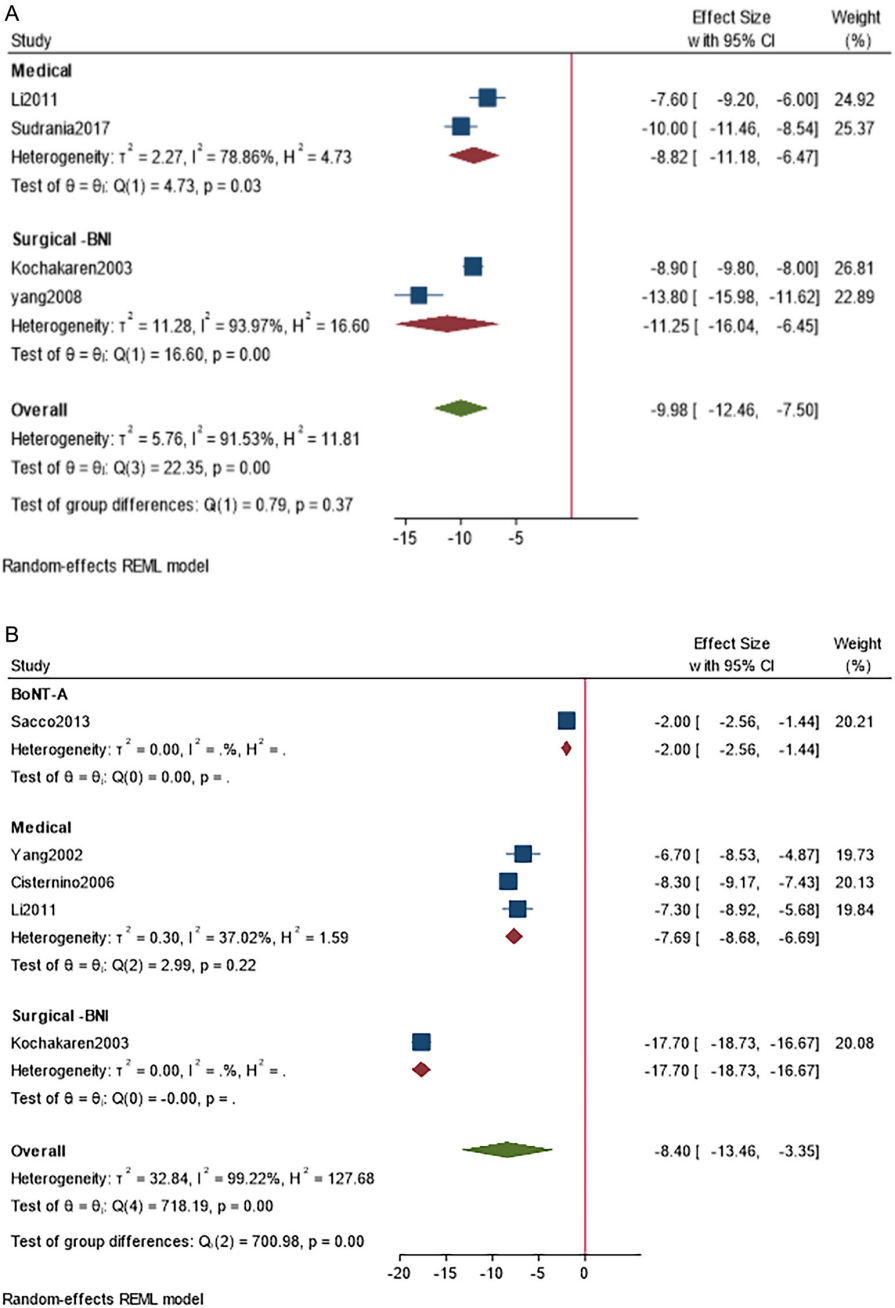
(A) Difference in IPSS between baseline and after 3 months. (B) Difference in IPSS between baseline and after 12 months. IPSS, International Prostate Symptom Score.

There is an 8.82 unit reduction in the IPSS score after 3 months of medical therapy from the baseline, whereas for the subgroup of surgical treatments, there is an 11.25 unit reduction in the IPSS score after 3 months from the baseline. The subgroup analysis reveals no significant difference in the reduction of IPSS between medical and surgical treatment subgroups (*P *= .37). The overall pooled estimate [irrespective of the subgroups] is found to be −9.98 [−12.46, −7.50], indicating that there is a 9.98 unit reduction in the IPSS score after 3 months from the baseline, which is found to be statistically significant (*P *< .001). Details are provided in Figure 2A.

There is a 7.69 unit reduction in the IPSS score after 12 months of medical therapy from the baseline, whereas for the subgroup of surgical treatments, there is a 17.70 unit reduction in the IPSS score after 12 months from the baseline (based on Kochakaren et al). The subgroup analysis reveals a significant difference in the reduction of IPSS between medical and surgical treatment subgroups (*P *< .001). The overall pooled estimate [irrespective of the subgroups] is found to be −8.40 [−13.46, −3.35], indicating that there is an 8.40 unit reduction in the IPSS score after 12 months from the baseline, which is found to be statistically significant (*P *< .001). Details are provided in Figure 2B.

#### 2. The Change in Q_max_ Between Baseline, 3 Months, and 12 Months:

**Figure 3:**
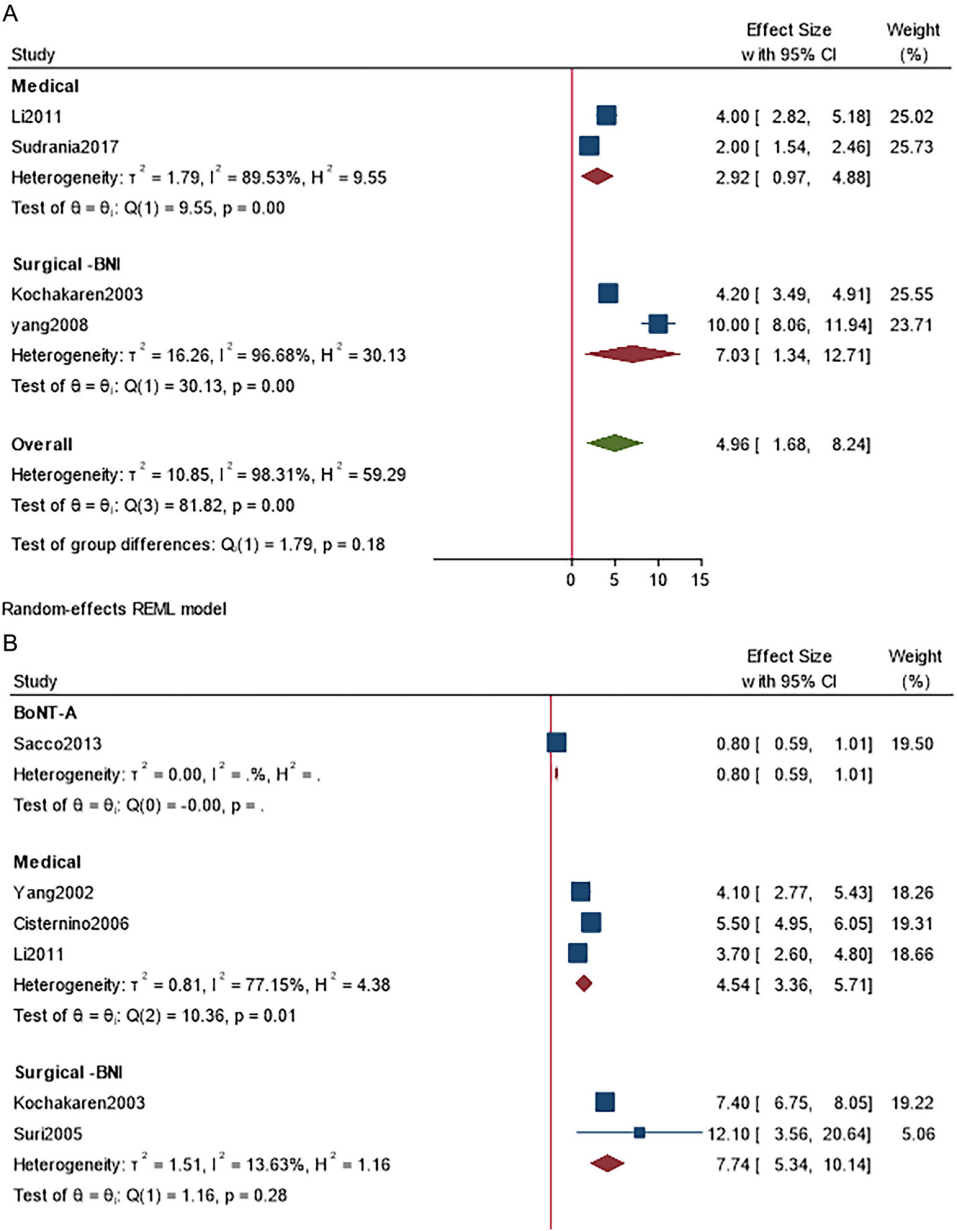
. (A) Difference in Q_max_ between baseline and after 3 months, (B) Difference in Q_max_ between baseline and after 12 months.

There is a 2.92 unit increase in the Q_max_ score after 3 months of medical therapy from the baseline, whereas for the subgroup of surgical treatments, there is a 7.03 unit increase in the Q_max_ score after 3 months from the baseline. The subgroup analysis reveals no significant difference in the increase of Q_max_ between the medical and surgical treatment subgroups (*P *= .18). The overall pooled estimate [irrespective of the subgroups] is found to be 4.96 [1.68, 8.24], indicating that there is a 4.96 unit increase in the Q_max_ score after 3 months from the baseline, which is found to be statistically significant (*P *< .001). Details are provided in Figure 3A.

There is a 4.54 unit increase in the Q_max_ score after 12 months of medical therapy from the baseline, whereas for the subgroup of surgical treatments, there is a 7.74 unit increase in the Q_max_ score after 12 months from the baseline. The subgroup analysis reveals a significant difference in the reduction of Q_max_ between medical and surgical treatment subgroups (*P *< .001). The overall pooled estimate (irrespective of the subgroups) is found to be 4.69 (2.46, 6.92), indicating that there is a 4.69 unit increase in the Q_max_ score after 12 months from the baseline, which is found to be statistically significant (*P *< .001). Details are provided in Figure 3B.

#### 3. The Change in Post-Void Residue Between Baseline, 3 Months, and 12 Months:

**Figure 4:**
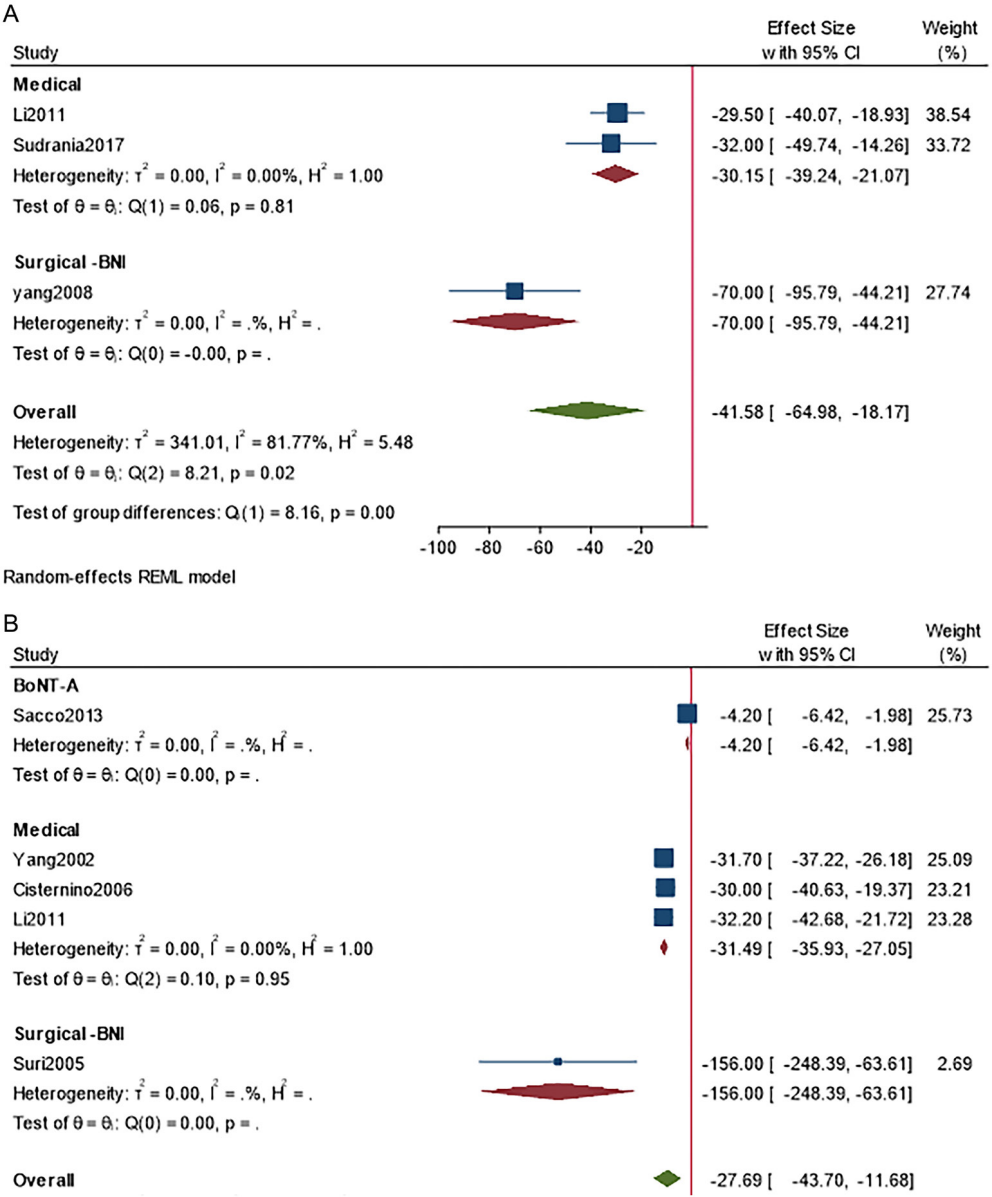
. (A) Difference in PVR between baseline and after 3 month, (B) Difference in PVR between baseline and after 12 months. PVR, post-void residue.

There is a 30.15 unit reduction in the PVR score after 3 months of medical therapy from the baseline, whereas for the subgroup of surgical treatments, there is a 70.00 unit reduction in the PVR score after 3 months from the baseline (based on Yang et al, 2008). The subgroup analysis reveals a significant difference in the reduction of PVR between the medical and surgical treatment subgroups (*P *< .001). The overall pooled estimate [irrespective of the subgroups] is found to be −41.58 [−64.98, −18.17], indicating that there is a 41.58 unit reduction in the PVR score after 3 months from the baseline, which is found to be statistically significant (*P *= .02). Details are provided in Figure 4A.

There is a 31.49 unit reduction in the PVR score after 12 months of medical therapy from the baseline, whereas for the subgroup of surgical treatments, there is a 156.00 unit reduction in the PVR score after 12 months from the baseline (based on Suri 2005 et al). The subgroup analysis reveals a significant difference in the reduction of PVR between the medical and surgical treatment subgroups (*P *< .001).

The overall pooled estimate [irrespective of the subgroups] is found to be −27.69 [−43.70, −11.68], indicating that there is a 27.69 unit reduction in the PVR score after 12 months from the baseline, which is found to be statistically significant (*P *< .001). Details are provided in Figure 4B.

## Discussion

Most of the men with PBNO present with mixed storage and voiding symptoms. Table 1 summarizes the various clinical presentations given in the studies. Voiding symptoms are usually more common than storage. While the IPSS score can rate the degree of bother, it cannot differentiate the chief symptom that is bothersome. This review noted long intervals of up to 10 years from initial symptoms to a precise vUDS diagnosis.^10^ While this might represent gradual disease progression, a diagnostic delay could be essential in some men. Of note, storage symptoms and pain can be factors confounding the diagnosis (Table 1). Pelvic pain or behavioral factors such as habitual or occupational postponement of voiding might be factors in other forms of voiding dysfunction, such as dysfunctional voiding. However, it remains uncertain whether these are important in young men with PBNO.^11^ Some of these men get repeated and prolonged antibiotics for presumed “chronic prostatitis.”^10^ Invasive vUDS evaluation that can provide a precise diagnosis should be considered in all men with refractory voiding difficulty to avoid delay in initiating appropriate therapy.

### Definition and Types

Most of the definitions of PBNO are the modifications in the definition used by Blaivas and Norlen in 1984, which stated PBNO as increased detrusor pressure during voiding, low uroflow with an obstructive flow pattern, narrowing of the bladder neck on fluoroscopic voiding cystourethrogram, and quietness of the external urethral sphincter on electromyography.^12^ The modifications include peak flow of fewer than 10 mL/s, detrusor pressure during voiding greater than 40 cm H_2_O, inadequate funneling of the bladder neck, opening pressure greater than 40 cm H_2_O with a relaxed external sphincter, postvoid residual urine volume of more than 100 mL, no associated neurologic defect, and a normal urethral caliber.^9^ Table 1 summarizes the definitions used in various studies to diagnose PBNO.

Nitti et al,^13^ described 3 types of PBNO: type 1 with high pressure and low flow voiding, type 2 with normal pressure and low flow and narrowing at the bladder neck, and type 3 with the delayed opening of the bladder neck. The clinical relevance of this classification has not been established. Paradoxically, young men under 30 years undergoing urodynamics are less likely to be obstructed and more likely to show an underactive detrusor.^14^

The diagnosis of PBNO through urodynamics relies on the proof of an obstruction in the bladder outlet and confirmation that the obstruction is located at the bladder neck. Pressure flow studies can clinch the diagnosis of obstruction and differentiate from underactivity of the detrusor. However, the applicability of the Bladder Outlet Obstruction Index and Bladder Contractility Index and their cutoffs that were developed for an older group of men is unclear.^15,16^

Diagnosis of the anatomical location of obstruction has traditionally relied on 2 different approaches. The first approach relies on demonstrating the silence of electromyographic signals from the pelvic floor and sphincter complex during voiding on either surface or (less commonly) needle electrode recording. The obstruction should be located at the bladder neck without any anatomical obstruction (which can confound this approach). The other method relies on a vvUDS demonstration of the failure to relax the bladder neck during voiding. Electromyographic recordings are not very reliable and can be confounded.^17^

This might explain why most investigators have chosen to use vUDS to make the diagnosis.

### Treatment Modalities

The various treatment modalities described are watchful waiting, adjunct behavioral therapy, pharmacological therapy, bladder neck incision, and injection of (OnaBoNT-A).

#### Watchful Waiting:

Watchful waiting can be tried in patients who have no significant bother. However, natural history has no evidence in cases of untreated PBNO patients. No case series or trials have been studied for this modality to date.^13^

#### Behavioral Therapy:

There is no evidence that biofeedback and behavioral therapy result in the improvement of voiding symptoms in PBNO. However, in patients presenting with infrequent volitional voiding, an attempt at timed voiding can be made. Although timed voiding, along with alpha-blocker therapy, is considered a better option.^18^ Behavioral therapy is a reasonable option for addressing the associated storage symptoms that some men report.^19^

#### Pharmacotherapy and Complications:

Alpha-blockers are the cornerstone of pharmacotherapy in PBNO. These relax the muscles of the bladder and neck. However, the alpha-blocker’s success rate is variable and not comparable to that of older men with presumptive benign prostatic obstruction.^20^ This review summarizes the impact of alpha-adrenergic blockers on IPSS, Q_max_, and PVR after 3 and 12 months. Although alpha-adrenergic blockers improve IPSS and Q_max _at 3 and 12 months, the results appear inferior to surgical treatment at 12 months. Most evidence suggests that alpha-adrenergic blockers have long-term efficacy.^21^

However, disease progression or changes in patients’ expectations in the medium term might result in lower satisfaction with medical therapy in some men. Studies have not explicitly been designed to study the comparative efficacy of medical and surgical management. The cohorts offered these 2 therapies are unlikely comparable, limiting conclusions concerning relative effectiveness. The various alpha-blockers used in studies are prazosin 2 mg twice daily, terazosin 5 mg at bedtime, doxazosin 1 or 2 mg at bedtime, alfuzosin 5 mg at bedtime, and tamsulosin 0.4 mg at bedtime.

The complication rate varies from 8%-61% in numerous studies in this review. The main complications were abnormal ejaculation, orthostatic hypotension, and dizziness.^18,22,23^

#### Bladder neck incision and complications:

The gold standard treatment for PBNO is the transurethral incision of the bladder neck, also known as bladder neck incision (BNI). The success rate achieved with this modality in long-term treatment is statistically more significant than with medical management.^9,24–26^ The classical procedure described is an incision from the neck of the bladder to the verumontanum at 5 o’clock or 7 o’clock. Various modifications have been given to decrease the complication rate associated with the surgery. The various modifications are:

1. Performing a unilateral BNI.
2. Preserving the supramontanal part of the prostate and extend the incision only 0.5-1 cm away from the verumontanum. Yang et al reported no cases of retrograde ejaculation with this technique.^25^
3. Suri et al performed BNI 2 mm proximal to the bladder neck at 12 o’clock untill verumontanum, without visualizing any fat.^9^
4. Mattiole et al^26^ used a thulium laser for bladder neck incision. They made an incision on the bladder neck at 7 o’clock from a line that connects the left ureteral opening to 1 cm above and to the side of the verumontanum.

Retrograde ejaculation and decreased sperm count are significant complications that were reported in 69%.^24^ The rate of ejaculatory dysfunction following the ejaculation-sparing technique was 0-0.02%.^26^ However, there is limited evidence in this regard. The method could be of critical importance to a younger group of men.

#### Onabotulinum Toxin A and Complications:

Onabotulinum toxin A recognizes and enters neurons via synaptic vesicle protein SV2, and they cleave synaptosomal-associated protein 25. This effect blocks neurotransmission as it inhibits the exocytosis of the neurotransmitter. There is no significant change in muscle fiber architecture in the human bladder with onaBoNT-A.^27,28^

Sacco et al^27^ used onaBoNT-A 200 U diluted in 4 mL of saline. This was injected into the bladder and neck. Although there was an improvement in IPSS, Q_max,_ and PVR until 9 months, the results were not statistically significant at 12 months. On average, the duration of action lasts for 8-11 months.

Complications associated with onaBoNT-A include urinary retention and painful micturition. There have been no reported cases of ejaculatory dysfunctions.^27^ Currently, the technique, dosage, and follow-up strategies are yet to be standardized, and efficacy has yet to be established by a broader set of investigators.

### The Change in Quality of Life

The mean QOL improved in both the medical and surgical therapy groups, as seen by the decrease in quality-of-life score data, although the data was highly variable among studies. Yang (2002) et al showed a drop in QOL score from 4.1 to 2.6 after 3 months of medical treatment, while Li et al showed a decrease in QOL score from 4.2 to 2.4 after 12 months of medical treatment, both of which were statistically significant in their respective studies. Similarly, Kochakaren et al and Yang 2008 et al studies significantly declined QOL scores after surgical treatment. This indicates that medical and surgical management resulted in a decline in QOL scores. Therefore, the improvement in QOL is seen in medical and surgical treatment groups. This suggests that the patient’s choice of therapy is critical, as there is no conclusive evidence of which therapy is superior based on subjective parameters.

### Disease Progression

It is currently unknown what happens in the natural progression of PBNO. Patients with severe obstructive flow may suffer long-term problems from delayed or ineffective diagnosis and treatment. Prolonged obstruction can result in reflux, hydroureteronephrosis, reduced detrusor muscle function, and eventual kidney damage.^29^

### Strengths and Limitations

Our study targets a clinically important but frequently understudied population by concentrating primarily on young adult males, providing findings that are immediately useful to urologists and other healthcare professionals who treat these patients. Our findings have more statistical power and generalizability due to the inclusion of several studies and the execution of a meta-analysis, allowing us to make recommendations for clinical practice based on solid data.

The definition of PBNO was not uniform across the studies. The definition of success was only based on a significant change in some studies. No definitive objective criteria were given for the success rate. Some studies did not report QOL scores for clinical interpretation. The studies had varying follow-up periods, but there was a lack of long-term data available. There was no comparison between various types of alpha-blockers. The various studies did not compare unilateral and bilateral bladder neck incisions. Men with voiding symptoms are most often and appropriately treated with an alpha-adrenergic blocker without resorting to invasive urodynamics. Responders are unlikely to be subjected to urodynamics and hence would never receive a precise urodynamic diagnosis. Such responders are also more likely to be men with voiding symptoms secondary to PBNO. This review would fail to capture such patients.

These shortcomings could be overcome by conducting a large multi-institutional randomized control trial analyzing different treatment modalities to establish the comparative efficacy and adverse events following each mode of care.

### Research Recommendations

This review identifies critical lacunae in evaluating and managing young men with PBNO. The authors would recommend the following areas for research: evaluation of the applicability of ICS Urodynamic Indices, Bladder Contractility Index and Bladder Outlet Obstruction Index to young men, and identification of prognostic cutoffs relevant to this age group; studies comparing alpha-adrenergic blockers and combination therapies of alpha-adrenergic blockers with overactive bladder medication; studies comparing ejaculation-sparing surgery with the current standard conventional bladder neck incision; studies examining different methods of performing a conventional bladder neck incision, such as unilateral, bilateral, and midline (6 o’clock); studies examining the long-term outcome of observation alone and medical and surgical therapies; and studies examining whether such men are at risk of requiring additional prostate surgery later in life.

## Conclusion

Primary bladder neck obstruction is a frequently diagnosed condition in young men experiencing LUTS. It requires a thorough assessment with vUDS and prompt intervention. While short-term success has been observed with alpha-blockers and onabotulinum toxin A, bladder neck incision has shown long-term efficacy based on objective criteria. The patient’s choice and subjective response rate should also be kept in mind before giving an appropriate treatment option to these patients.

## Figures and Tables

**Table 1.** Overall Characteristics and Characteristics of Individual Studies

**Serial No. **	**Author **	**Age (Range/SD)**	**Type of Study**	**No. of Patients**	**Presenting Symptoms **	**Technique of UDS**	**Definition of PBNO Used in the Study**	**Duration of Symptoms (Months) (SD/Range)**
1	Yang, 2002	39.3 ± 7.4	Prospective	28	Obstructive	vUDS	Relaxed external sphincter electromyography during voiding, no distal urethral obstruction, and narrowing only at the vesical neck on voiding cystourethrography under fluoroscopic guidance	18 ± 11.6
2	Suri, 2005	33.42 ± 6.56	Retrospective	45	Obstructive	vUDS	Peak flow less than 10 mL/s, detrusor pressure during voiding greater than 40 cm H_2_O, inadequate funneling of the bladder neck, opening pressure greater than 40 cm H_2_O with a relaxed external sphincter and postvoid residual urine volume of more than 100 mL, no associated neurologic defect, and a normal urethral caliber.	45.6 ± 49.2
3	Cisternino, 2006	43 (31-49)	Retrospective	41	Frequency, nocturia, weak stream	vUDS	NA	18 (6-20)
4	Li, 2011	27.3 (18-35)	Retrospective	24	Hesitancy, weak stream, frequency	vUDS	Radiographic evidence of inadequate funnelling of the bladder neck with relaxation of the external sphincter during voiding and no distal urethral obstruction, detrusor pressure at maximum flow greater than 20 cm H_2_O and maximum flow rate (Q_max_) of less than 15 mL/s	26.4 (3-65)
5	Sudrania, 2017^12^3	41 (18-50)	Prospective	21	LUTS	vUDS	NA	50
6	Kaplan, 1994	38.5 (26-51)	Retrospective	31	Obstructive, irritative LUTS, perineal pain	vUDS	Bladder outlet obstruction was defined as patients with a maximal urine flow of less than 15 mL. per second and detrusor pressure at a maximal flow of greater than 45 cm H_2_O. Fluoroscopy was used to determine the anatomical site of obstruction or narrowing.	38.3 (25-126)
7	Kochakaren, 2003	40.3 ± 2.41	Retrospective	35	NA	NA	NA	NA
8	Suri, 2005	33.42 ± 6.56	Retrospective	18	Obstructive	vUDS	Peak flow less than 10 mL/s, detrusor pressure during voiding greater than 40 cm H_2_O, inadequate funneling of the bladder neck, opening pressure greater than 40 cm H_2_O with a relaxed external sphincter and postvoid residual urine volume of more than 100 mL, no associated neurologic defect, and a normal urethral caliber.	45.6
9	Yang, 2008	41.9 ± 6.9	Prospective	26	Storage and voiding LUTS	vUDS	Narrowing only at the bladder neck on voiding cystourethrography, sustained detrusor contraction during voiding with detrusor pressure 30 cm H_2_O, maximum flow rate—15 mL/s, relaxed external sphincter electromyography during voiding and no distal urethral obstruction radiologically.	26.1 (19.4)
10	Mattioli, 2016	42 (21-45)	Prospective	196	Storage and voiding LUTS	vUDS	NA	NA
11	Sacco, 2013	33.8 (19-48)	Prospective	30	LUTS	vUDS	NA	55.2 (12-84)

UTS, lower urinary tract symptoms; NA, not available; PBNO, primary bladder neck obstruction; vUDS, video urodynamics.

**Table 2.** Treatment Options Used in Different Studies and Subgroups

**Serial No. **	**Author **	**Previously Failed Treatment**	**Alpha-Blockers Used/BNI Surgery/Onabotulinum Toxin**	**Group**	**Adverse Events**	**Definition of Success**	**Successful**	**Follow-up (Months)**
1	Yang, 2002	3	Doxazosin 1 mg or 2 mg bedtime for 3 months	Medical	None reported by patients.	IPSS decrease 50%, Q_max_ increase >2.5 mL/s	46%	12
2	Suri, 2005		Prazosin or terazosin	Medical	Orthostatic hypotension—2 patients	IPSS, Q_max_ (if significant change)	48%	96
3	Cisternino, 2006		Alfuzosin 5 mg, Tamsulosin 2 mg	Medical	None	AUA score, Q_max_ and PVR if significantly changed	70.7%	12
4	Li, 2011		Doxazosin 4 mg	Medical	2 patients had dizziness, Somnolence	IPSS, Q_max _(if significant change)	66.6%	16.3
5	Sudrania, 2017		Tamsulosin 0.4 mg	Medical	Abnormal ejaculation—10, Giddiness—3	Decrease in PdetQ_max_ by 15%	57%	3
6	Kaplan, 1994	Failed alpha-blockers	BNI 5 o’clock	Surgical—BNI	No RE	Symptoms score, Q_max _(if significant change)	96%	7.3
7	Kochakaren, 2003		BNI Unilateral	Surgical—BNI	Sperm count decreased 69%	IPSS, Q_max _(if significant change)	100%	12
8	Suri, 2005	Failed alpha-blockers	BNI Unilateral 12 o’clock	Surgical—BNI		IPSS, Q_max _(if significant change)	100%	96
9	Yang, 2008	Failed alpha-blockers	BNI Modified Bilateral 5 and 7 o’clock—Nd-Yag laser	Surgical—BNI	No RE	IPSS decrease 25%, Q_max_ increase >2.5 mL/s	84%	24
10	Mattioli, 2016	3	BNI unilateral—157, B/L—39 using Thulium laser	Surgical -BNI	RE—3 patients, Decreased semen volume—14	IPSS, Q_max _(if significant change)	NA	3
11	Sacco, 2013		Onabotulinum toxin A—200 U at 3, 6, 9, 12 o’ clock	BoNT-A		IPSS, Q_max _(if significant change)	NA	11.4

AUA score, American Urological Association Symptom Score; B/L, bilateral; BNI, bladder neck incision; BoNT-A, onabotulinum toxin A; IPSS, International Prostate Symptom Score; NA, not available; PVR, post-void residue; Q_max_, maximum flow rate; RE, retrograde ejaculation.

**Table 3.** Quality of Life Score in Various Studies

**Serial No. **	**Author **	**QOL Pretreatment **	**QOL Post 3mo**	**QOL Post 6 month**	**QOL Post 12 month**
1	Yang, 2002	4.1	2.6	NA	NA
2	Suri, 2005	NA	NA	NA	NA
3	Cisternino, 2006	NA	NA	NA	NA
4	Li, 2011	4.2	NA	NA	2.4
5	Sudrania, 2017	5	4	NA	NA
6	Kaplan, 1994	NA	NA	NA	NA
7	Kochakaren, 2003	5	NA	NA	1
8	Suri, 2005	NA	NA	NA	NA
9	Yang, 2008	4.2	2	NA	NA
10	Matioli, 2016	NA	NA	NA	NA
11	Sacco, 2013	5	NA	2.6	4.9

NA, not available; QOL, quality of life.

**Supplementary Table 1.** Search Strategy for Selection of Eligible Studies to be Included in the Systematic Review

Serial No	Database	Search items	Number of studies identified
1.	PubMed	Primary bladder neck obstruction OR Primary bladder neck dysfunction OR Bladder neck obstruction NOT women NOT children NOT females	3346
2.	Embase	Primary bladder neck obstruction OR Primary bladder neck dysfunction OR Bladder neck obstruction NOT women NOT children NOT females	236
3.	Cochrane	Primary bladder neck obstruction OR Primary bladder neck dysfunction OR Bladder neck obstruction NOT women NOT children NOT females	0

**Supplementary Table 2.** Methodological Index for Non-randomized Studies (MINORS) Bias Assessment

**Author **	**Clearly stated aim**	**Inclusion of consecutive patients **	**Prospective collection of data**	**Endpoints appropriate to the aim of the study **	**Unbiased assessment of the study endpoints **	**Prospective calculation of study sample**	**Total MINORS score**	**Oxford level of evidence (Separate from MINORS score)**
Yang2002	2	2	2	2	2	0	10	2B
Suri2005	2	0	0	2	2	0	6	4
Cisternino2006	2	2	0	2	2	0	8	4
Li2011	2	0	0	2	2	0	6	4
Sudrania2017	2	2	2	2	2	0	10	2B
Kaplan1994	2	2	2	2	2	0	10	2B
Kochakaren2003	2	2	2	2	2	0	10	2B
yang2008	2	2	2	2	2	0	10	2B
Mattioli2016	2	0	0	2	2	0	6	2B
Sacco2013	2	2	2	2	2	0	10	2B
**MINORS scale interpretation: **3								
Not reported	0	3	3	3	3	3	3	3
Reported but inadequate	1	3	3	3	3	3	3	3
Reported and adequate	2	3	3	3	3	3	3	3

**MINORS >50% is considered high quality study.**
